# Enhanced lipid metabolism induces the sensitivity of dormant cancer cells to 5-aminolevulinic acid-based photodynamic therapy

**DOI:** 10.1038/s41598-021-86886-9

**Published:** 2021-03-31

**Authors:** Taku Nakayama, Tomonori Sano, Yoshiki Oshimo, Chiaki Kawada, Moe Kasai, Shinkuro Yamamoto, Hideo Fukuhara, Keiji Inoue, Shun-ichiro Ogura

**Affiliations:** 1 Center for Photodynamic Medicine, Kochi Medical School, Kohasu, Oko-cho, Nankoku-shi, Kochi 783-8505 Japan; 2 School of Life Science and Technology, Tokyo Institute of Technology, 4259 Nagatsuta-cho, Midori-ku, Yokohama, Kanagawa 226-8501 Japan; 3 Kochi Medical School, Kohasu, Oko-cho, Nankoku-shi, Kochi 783-8505 Japan; 4 Tazuke Kofukai Medical Research, Institute Kitano Hospital, Osaka, Japan; 5 Department of Urology, Kochi Medical School, Kohasu, Oko-cho, Nankoku-shi, Kochi 783-8505 Japan

**Keywords:** Cancer metabolism, Cancer models, Urological cancer, Cancer

## Abstract

Cancer can develop into a recurrent metastatic disease with latency periods of years to decades. Dormant cancer cells, which represent a major cause of recurrent cancer, are relatively insensitive to most chemotherapeutic drugs and radiation. We previously demonstrated that cancer cells exhibited dormancy in a cell density-dependent manner. Dormant cancer cells exhibited increased porphyrin metabolism and sensitivity to 5-aminolevulinic acid-based photodynamic therapy (ALA-PDT). However, the metabolic changes in dormant cancer cells or the factors that enhance porphyrin metabolism have not been fully clarified. In this study, we revealed that lipid metabolism was increased in dormant cancer cells, leading to ALA-PDT sensitivity. We performed microarray analysis in non-dormant and dormant cancer cells and revealed that lipid metabolism was remarkably enhanced in dormant cancer cells. In addition, triacsin C, a potent inhibitor of acyl-CoA synthetases (ACSs), reduced protoporphyrin IX (PpIX) accumulation and decreased ALA-PDT sensitivity. We demonstrated that lipid metabolism including ACS expression was positively associated with PpIX accumulation. This research suggested that the enhancement of lipid metabolism in cancer cells induces PpIX accumulation and ALA-PDT sensitivity.

## Introduction

5-aminolevulinic acid (5-ALA) is an amino acid that is a precursor for the biosynthesis of porphyrins in plants and animals, and porphyrins biosynthesized from 5-ALA function as important cofactors in plant and animal cells. Various metals are inserted into the structure of porphyrins, and these metal porphyrins have several functions in vivo. For example, magnesium ions inserted in porphyrins are important constituents of chlorophyll, and iron ions inserted in porphyrins are important constituents of heme, which functions as an active site for various enzymes such as CYP450, catalase, and mitochondrial electron transport chain complexes^1,2^. Protoporphyrin IX (PpIX) is a fluorophore with a maximum excitation wavelength of about 405 nm and a maximum emission wavelength of about 635 nm; metal-free porphyrins, including PpIX, function as photosensitizers. Furthermore, various types of tumors have been found to accumulate PpIX after ALA treatment although the mechanism is unknown^1–5^. Therefore, ALA has been used clinically for the photodynamic diagnosis (ALA-PDD) and treatment (ALA-PDT) of malignant tumors^6^.

Although ALA-PDD and ALA-PDT are widely used clinically, the mechanism of PpIX accumulation and the differences between cancer cells with high and low PpIX accumulation remain unclear. We identified the ATP-binding cassette transporter ABCG2 and peptide transporter PEPT1 as key regulators of intracellular PpIX levels in vitro and in bladder cancer specimens^7^. Moreover, we clarified the effects of plasma membrane ABCB6 levels on porphyrin accumulation under hypoxia^8^. Another report uncovered that ABCB6 upregulation was critical for PpIX accumulation^9^. Altogether, these results suggest that PEPT1, ABCB6, and ABCG2 are critically involved in porphyrin metabolism^1,2^.

The latency period for cancer to recur can span years or even decades^10,11^. This delay can be explained by cancer dormancy^10^. Dormant cancer cells are relatively insensitive to most chemotherapeutic drugs and radiation. The cells can cause tumor recurrence when they re-enter the cell cycle^2,10,12,13^. Antonija et al. followed the repopulation dynamics of 150 single lentivirus-marked lineages from 10 human colorectal cancers through serial xenograft passages in mice, obtaining evidence of a relatively dormant or slowly proliferating cell population in primary human colorectal cancer cells that retains potent tumor propagation potential, thereby preferentially driving tumor growth after chemotherapy^14^. We previously demonstrated that cancer cells exhibited dormancy in a cell density-dependent manner. Cancer dormancy was characterized by no proliferation, no death, metabolic suppression, and active status recovery^15^. The high-density 2D and 3D culture model, as a dormant cancer cells model, exhibited strong PpIX accumulation and sensitivity to ALA-PDT. In dormant cancer cells, PEPT1 and ABCB6 were upregulated, and ABCG2 was downregulated. PpIX accumulation and ALA-PDT cytotoxicity were enhanced by G0/G1-phase arrestors in non-dormant cancer cells^2^. In addition, heme levels were also increased in dormant cancer cells that had not been exposed to ALA^16^. These results suggest that porphyrin metabolism was enhanced in those cells. In this study, we conducted microarray analysis of human mRNA in PC-3 prostate cancer cells to reveal metabolic changes caused by cellular dormancy and clarify their effects on sensitivity to ALA-PDT.

## Methods

### Biochemicals

ALA hydrochloride was procured from SBI Pharmaceuticals Co., Ltd. (Tokyo, Japan). We obtained DMEM from Thermo Fisher Scientific (Waltham, MA, USA). Antibiotic antimycotic (ABAM) and trypan blue stain solutions were purchased from Nacalai Tesque (Kyoto, Japan). Fetal bovine serum (FBS) was purchased from Thermo Fisher Scientific. Triacsin C was purchased from Cayman Chemical (Ann Arbor, MI, USA). All reagents used in this research were of the highest purity available.

### Cells and cell cultures

The human prostate cancer cell line PC-3 (provided by Dr. Inoue, Kochi University, Kochi, Japan) was maintained in DMEM supplemented with 10% (v/v) FBS and 1% (v/v) ABAM. Cells were maintained in a 5% CO_2_ atmosphere at 37 °C^1^.

### 3D cell culture

EZSPHERE 3D cell culture plates were obtained from AGC Techno Glass Co., Ltd. (Tokyo, Japan) and used to culture cancer spheroids. In total, 5 × 10^5^ (S500) or 1.25 × 10^5^ cells (S125) were seeded with 3 ml of medium in each 35-mm dish. After 2 days, 1 ml of old medium was carefully replaced with fresh medium. Four days after seeding, 2300 spheroids were formed in each dish^2^.

### Analysis of PpIX accumulation

Intracellular PpIX levels were determined using a confocal microscope or microplate reader. Cells were treated with triacsin C alone for 48 h, followed by co-culture with triacsin C and ALA for 24 h. Extracellular PpIX was removed via washing by PBS before analysis. An FV-1000D downright laser-scanning confocal microscope (Olympus, Tokyo, Japan) was used for live-cell microscope imaging. The excitation wavelength was set at 405 nm for PpIX and 635 nm for DRAQ5 (BioStatus, Loughborough, United Kingdom). The emission wavelength was set at 560–800 nm for PpIX and 655–755 nm for DRAQ5. Laser illumination was set at 3.0% power for PpIX and 5.0% power for DRAQ5. All images were acquired using a × 60 oil immersion lens. The images were analyzed using Olympus Fluoview ver. 4.2b software. A Cytation 5 microplate reader (BioTek, Winooski, VT, USA) was used for the live-cell relative quantification of intracellular PpIX content. The excitation wavelength was set at 385–425 nm for PpIX and 330–370 nm for Hoechst 33,342 (Thermo Fisher Scientific). The emission wavelength was set at 615–655 nm for PpIX and 430–470 nm for Hoechst 33,342. Fluorescence was measured at 10 points in each well.

### Quantitative PCR

Total cellular RNA was purified using an RNeasy Mini kit (QIAGEN, Hilden, Germany). Further, cDNA was generated via reverse transcription using a SuperScript III First-Strand Synthesis System for RT-PCR (Invitrogen, Thermo Fisher Scientific). Quantitative PCR was performed using the StepOnePlus Real-Time PCR System (Applied Biosystems, Thermo Fisher Scientific)^1^. TaqMan probes were selected for PCR (Ki-67, Hs04260396-g1; MCM7, HS00428518-m1; PEPT1, Hs00953898-m1; ABCB6, HS01039213-m1; ABCG2, Hs01053790-m1; ActB, HS01060665-g1). All procedures were performed according to the manufacturer’s instructions.

### Microarray

The quality of purified total cellular RNA was measured at 260 and 280 nm before microarray analysis. We confirmed that the RNA integrity number of all RNA samples was at least 9.50. The Clariom S Assay for humans (Thermo Fisher Scientific) was applied for all samples to analyze human mRNA expression. The raw result data were analyzed using Transcriptome Analysis Console ver. 4.0.1.36. The significance of log fold change (logFC) values for RNAs was evaluated using *t*-tests, and the *p* values associated with logFC values were adjusted for multiple testing using the false discovery rate (FDR)^17,18^. Significantly different expression was indicated by FDR-adjusted *p* < 0.05.

### Light irradiation

Cells were incubated with ALA at 37 °C in an atmosphere of 5% CO_2_ for 24 h and exposed to light-emitting diode (LED) irradiation at an intensity of 14.2 mW/cm^2^ (635 nm) for 5 min by placing the plate below the LED irradiation unit (Bio Research Center, Nagoya, Japan) as previously described^19^. Cells were further incubated in the dark overnight, and their viability was then measured using the MTT cell proliferation assay as previously described^2^.

### Statistical analysis

Microsoft Office Excel software was used for statistical analysis and graph preparation. An unpaired two-tailed *t*-test was used to test the significance of differences between groups. The data were expressed as the mean ± SE of at least three independent experiments^1^.

### Ethical approval

This research does not include direct participation of any human/animals. Hence, ethics committee approval, informed consent to participate/publish is not applicable here.

## Results

### Microarray mRNA analysis overview

**Figure 1:**
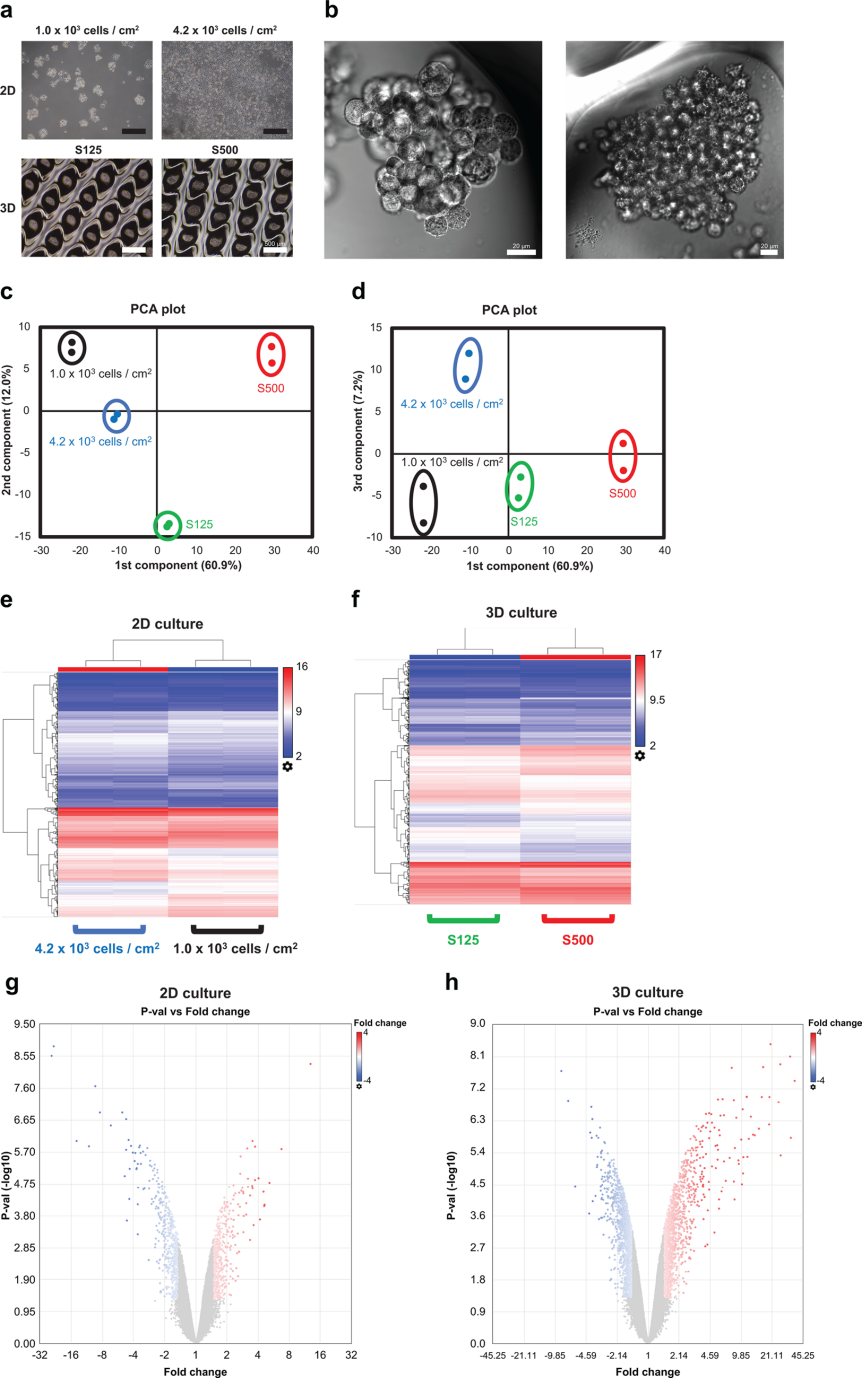
Microarray profiles of mRNA expression. All data were calculated using Transcriptome Analysis Console ver. 4.0.1.36. (**a**) Phase contrast images of 2D cultured cells and 3D cultured spheroids. The scale bar is 500 μm. (**b**) Differential interference contrast images of 3D cultured spheroids. The scale bar is 20 μm. (**c**) A 2D principal component analysis (PCA) plot of the first and second components. (**d**) A 2-D PCA plot of the first and third components. (**e**,**f**) 2D and 3D cluster analysis of differentially expressed genes. Red indicates increased expression, and blue denotes decreased expression. (**g**,**h**) The volcano plot of differentially expressed genes in 2D and 3D culture. Red indicates increased expression, and blue denotes decreased expression.

We constructed 2D and 3D cultures with different dormant statuses as described in our previous research^2^. The culture time was 4 days for all conditions. In the 2D culture before analysis, a density of 1.0 × 10^3^ cells/cm^2^ represented 20% confluency, and a density of 4.2 × 10^3^ cells/cm^2^ represented 80% confluency. The spheroid diameter was 125 μm for S125 and 180 μm for S500 (Fig. 1a,b). In short, we revealed that cellular dormancy had a positive relationship with cell density on PC-3 cells^2^. The principal component analysis (PCA) plot confirmed that the first component had a proportional relationship with cell dormancy. However, the second and third components were unclear (Fig. 1c,d). The heat map images and volcano plots for both the 2D and 3D cultures visualized differentially expressed mRNAs (Fig. 1e–h). All mRNA expression results in microarray are presented in Supplementary Table S1.

### Pathway analysis according to the dormancy status

**Figure 2:**
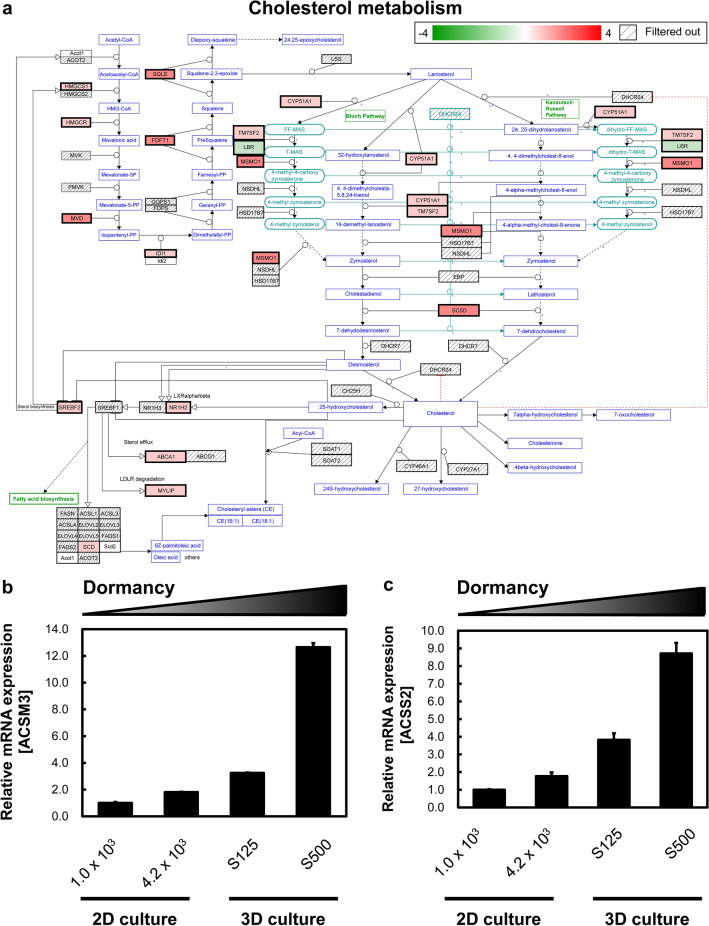
Lipid metabolisms change in dormant cancer cells. (**a**) Cholesterol metabolism involved in 3D cultured S500 and S125 spheroids. S500 spheroids displayed higher cholesterol metabolism than S125 spheroids. Genes were selected from the “Cholesterol metabolism (includes both Bloch and Kandutsch–Russell pathways) (Homo sapiens)” pathway according to Wikipathways. Red indicates increased expression, and green denotes decreased expression. (**b**) The mRNA expression of acyl-CoA synthetase medium chain family member 3. (**c**) The mRNA expression of acyl-CoA synthetase short-chain family member 2. All mRNA expression analyses were conducted using Transcriptome Analysis Console ver. 4.0.1.36. Genes with changes in expression exceeding 1.5-fold and significant at *p* < 0.05 genes were extracted. n = 2.

We next analyzed metabolic pathway changes in different cellular dormancy models. Interestingly, lipid-related pathways were upregulated in dormant cancer cells. For example, the cholesterol metabolism map revealed that dormant cancer cells exhibited greater cholesterol synthesis than non-dormant cancer cells (Fig. 2a). In addition, the lipid metabolism-related genes acyl-CoA synthetase medium chain family member 3 and acyl-CoA synthetase short-chain family member 2^20,21^ were dramatically upregulated in a dormancy-dependent manner (Fig. 2b,c). Therefore, we conducted further analysis of the relationship between lipid metabolism and porphyrin metabolism after ALA administration in 2D culture.

### Triacsin C cytotoxicity in PC-3 cells

**Figure 3:**
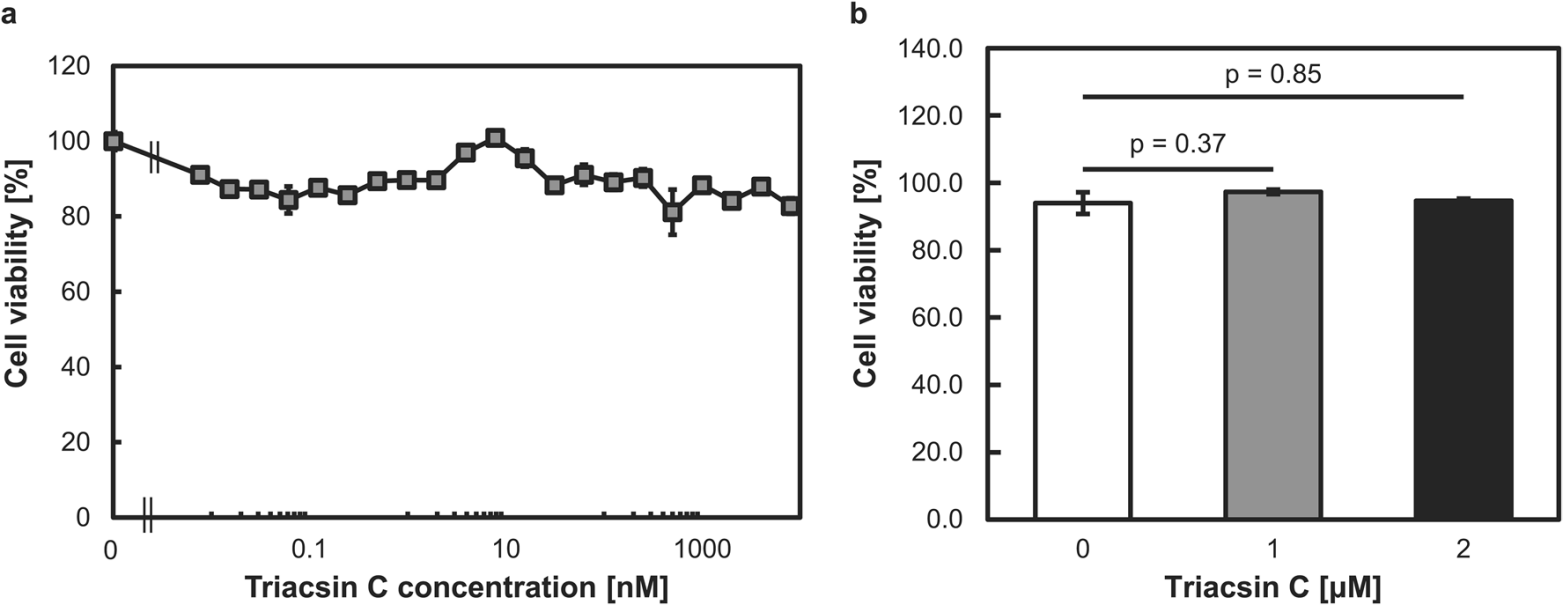
Effects of triacsin C on PC-3 cell proliferation. Cells were treated with various concentrations of triacsin C for 72 h. (**a**) Cell viability was determined using the MTT assay. n = 4. (**b**) Cell viability was determined using the trypan blue stain. n = 3. Bars represent the SE.

Triacsin C is a potent inhibitor of acyl-CoA synthetases (ACSs), which act downstream of FASN and convert long-chain fatty acids to acyl-CoA^22^. This reaction is a crucial step in several lipid metabolism pathways, including phospholipid biosynthesis, lipid modification of cellular proteins, and β-oxidation. In mammals, five ACS isozymes have been identified. Previous reports indicated that ACSs suppress apoptosis and that ACS inhibition by triacsin C could be a rational strategy for amplifying the antitumor effect of etoposide^22,23^. In this study, we selected triacsin C as the lipid metabolism suppressor for further study. Triacsin C at concentrations of up to 8 μM did not induce cytotoxicity in PC-3 cells after 72 h of exposure (Fig. 3a). Thus, we selected triacsin concentrations of 1 and 2 μM for further porphyrin-related analyses (Fig. 3b).

### Porphyrin-related transporter expression changes following triacsin C exposure

**Figure 4:**
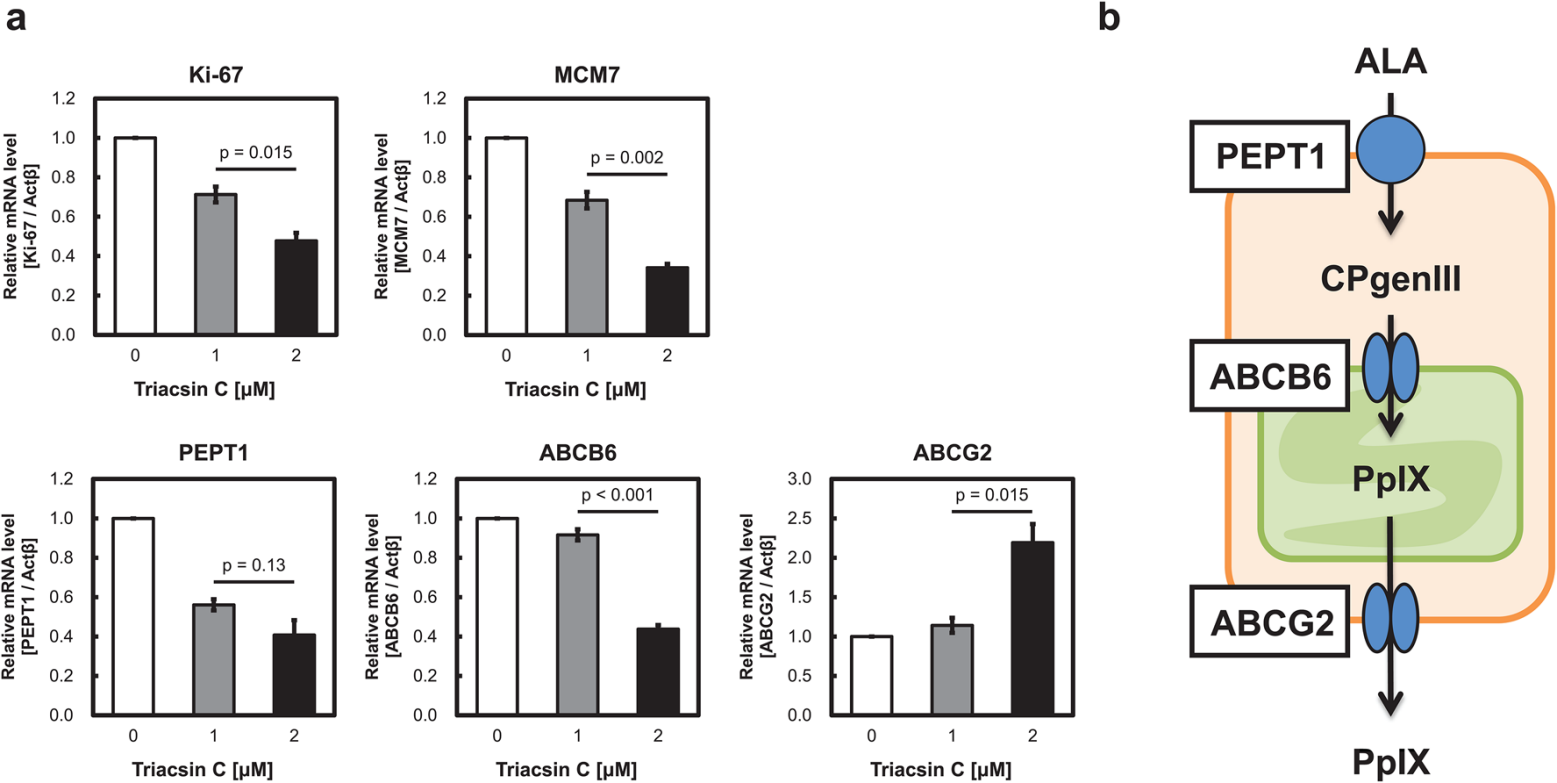
mRNA expression changes induced by triacsin C treatment. (**a**) The mRNA expression levels of the cell proliferation markers Ki-67 and MCM7 and porphyrin-related transporters PEPT1, ABCB6, and ABCG2 were measured. The cells were treated with triacsin C for 72 h. n = 3. Bars represent the SE. (**b**) Dominant transporters involved in porphyrin metabolism.

We investigated the expression of transporters involved in porphyrin metabolism. We previously revealed that PEPT1, ABCB6, and ABCG2 expression was remarkably changed in dormant cancer cells, leading to increased porphyrin metabolism^2^. Specifically, the expressions of PEPT1 and ABCB6 were upregulated in dormant cancer cells, whereas that of ABCG2 was downregulated. Ki-67 and MCM7, which are cell proliferation markers, were downregulated by triacsin C treatment because of its antitumor activity^24^. The mRNAs of PEPT1 and ABCB6 expressions were decreased after 72 h of triacsin C treatment, whereas that of ABCG2 was upregulated (Fig. 4a). These transporter expression changes suggest that low PpIX accumulation was induced by triacsin C (Fig. 4b). These results were consistent with our previous reports.

### Triacsin C reduced PpIX accumulation after ALA administration

**Figure 5:**
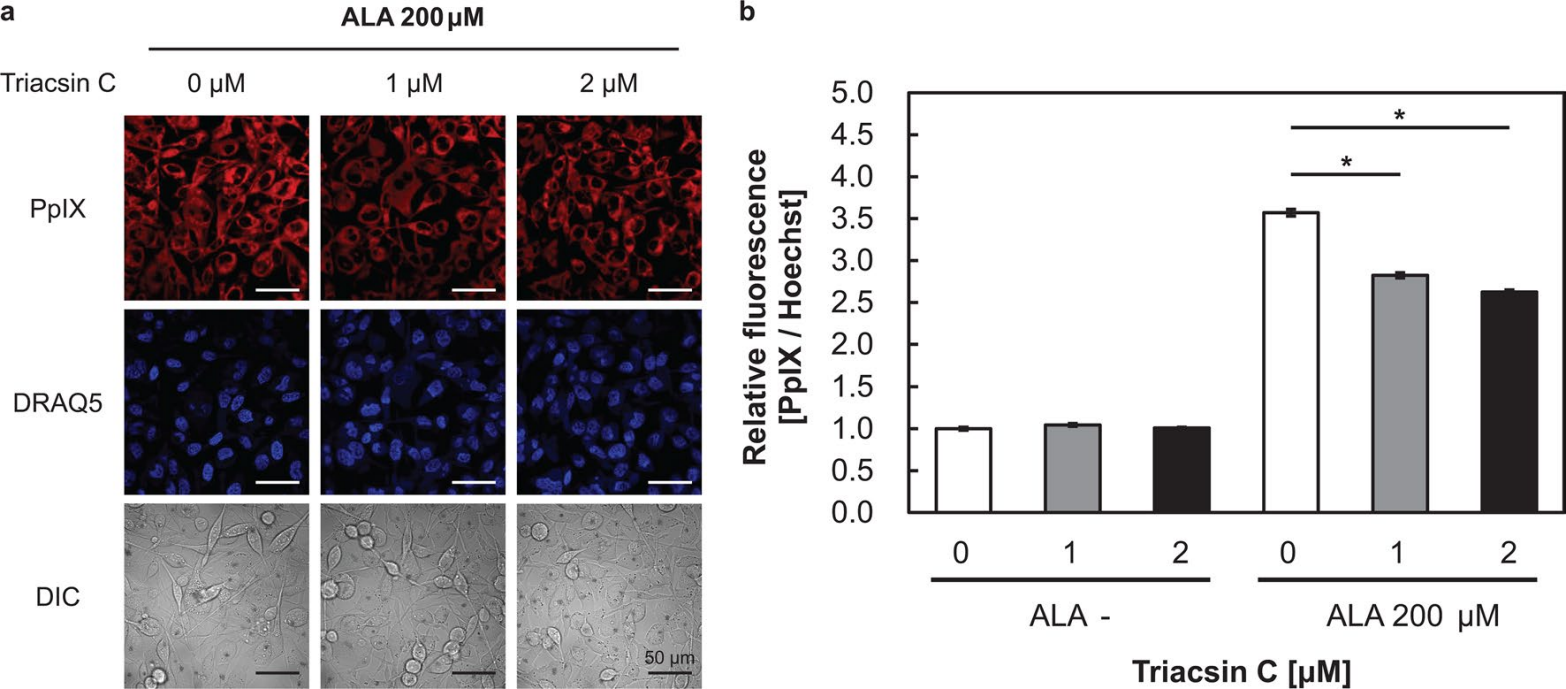
Triacsin C suppressed protoporphyrin IX (PpIX) accumulation after 5-aminolevulinic acid (ALA) treatment. Cells were treated with triacsin C alone for 48 h and then co-cultured with triacsin C and ALA for 24 h. (**a**) Confocal laser-scanning microscopy images of DRAQ5 (nuclei) and PpIX. Scale bar, 50 μm. (**b**) PpIX accumulation in the presence of absence of triacsin C as measured using a microplate reader. n = 3. *p < 0.001. All bars represent the SE.

Next, we investigated PpIX accumulation after triacsin C and ALA exposure. Confocal microscopy revealed that the fluorescence intensity of PpIX was decreased by triacsin C treatment (Fig. 5a). Live-cell relative quantification analysis also supported the decreased PpIX content following triacsin C treatment (Fig. 5b). These results are consistent with the results of transporter expression in Fig. 4.

### ALA-PDT cytotoxicity was decreased by triacsin C

**Figure 6:**
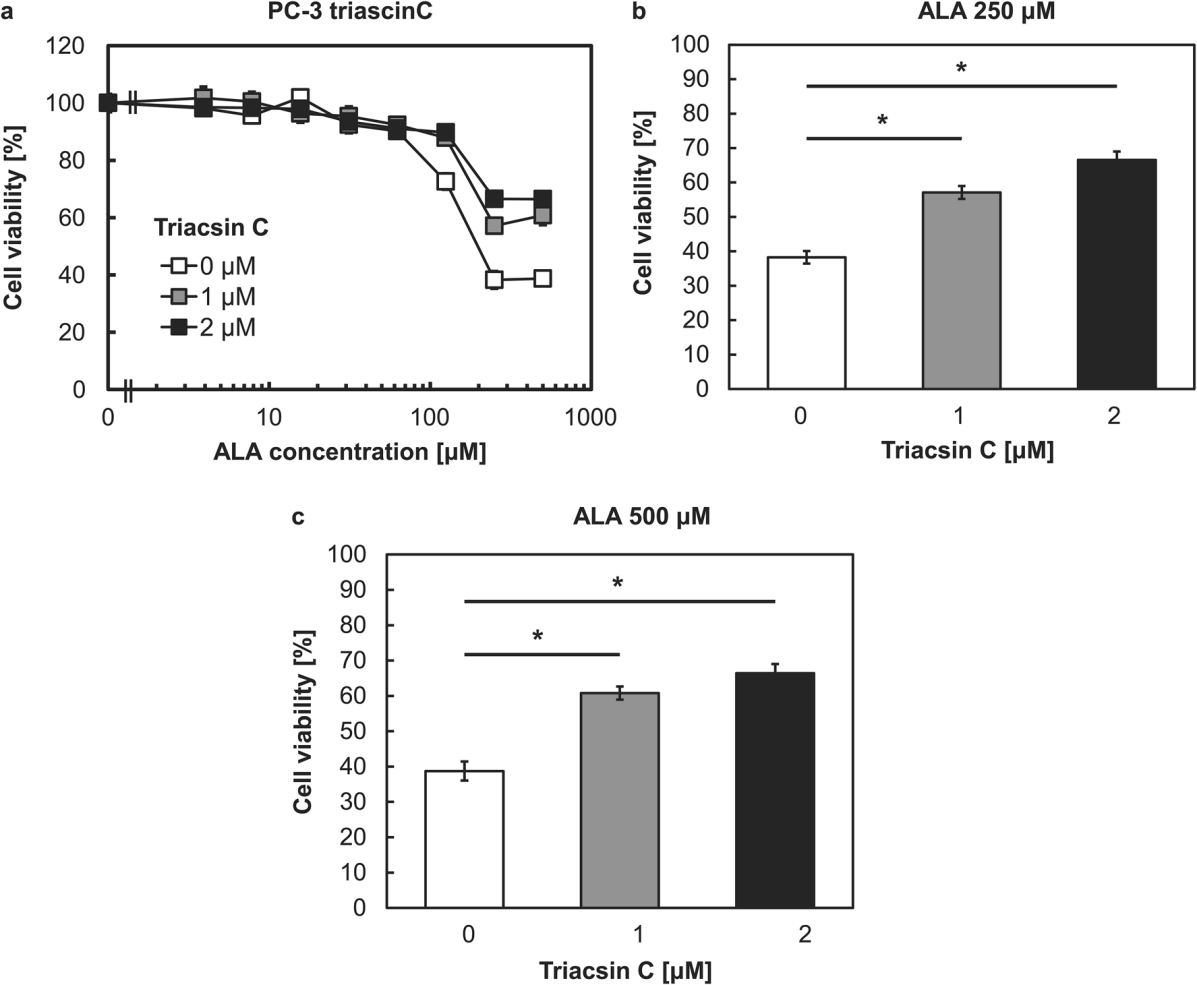
Cell viability after 5-aminolevulinic acid-based photodynamic therapy (ALA-PDT) with or without triacsin C. Cells were treated with triacsin C alone for 48 h and then co-cultured with triacsin C and ALA for 24 h. Cells were irradiated with 14.2 mW/cm^2^ light for 5 min. Cell viability was determined on the next day using the MTT assay. (**a**) Cell viability in the presence of 0–2 μM Triacsin C and various ALA concentrations. (**b**) Cell viability in the presence of 250 μM ALA. (**c**) Cell viability in the presence of 500 μM ALA. n = 8. *p < 0.001. All bars represent the SE.

ALA-PDT can lead to cell death via necrosis or apoptosis, and it is a highly effective form of therapy for treating superficial basal cell carcinomas. To investigate the cytotoxicity after photoirradiation with triacsin C, we irradiated cells with red light at an intensity of 14.2 mW/cm^2^ for 5 min. Triacsin C increased cell viability in the presence of ALA concentrations exceeding 125 μM in a concentration-dependent manner (Fig. 6a). Specifically, 2 μM triacsin C increased cell viability by 30% in the presence of 250 and 500 μM ALA (Fig. 6b,c). Although triacsin C has an absorbance peak at 301 nm, it will not be a photosensitizer to generate fluorescence or ROS since ALA-PDT’s photoirradiation was performed at 635 nm. These results indicated that the cytotoxicity of ALA-PDT has a positive relationship with lipid metabolism.

## Discussion

In this study, we first demonstrated metabolic differences between dormant and non-dormant cancer cells via microarray analysis. Metabolism pathway analysis revealed specific changes in lipid metabolism in dormant cancer cells. Second, we demonstrated that PpIX accumulation was decreased by exposure to triacsin C, a key inhibitor of lipid metabolism. Moreover, ALA-PDT cytotoxicity was decreased by triacsin C treatment. To the best of our knowledge, this is the first study to demonstrate that lipid metabolism affects PpIX accumulation and ALA-PDT therapeutic efficacy.

Cancer cells are often exposed to a metabolically challenging environment with scarce availability of oxygen and nutrients. This metabolic stress leads to changes in the balance between the endogenous synthesis and exogenous uptake of fatty acids, which are required by cells for membrane biogenesis, energy production pathways including the TCA cycle in mitochondria, and protein modification^25,26^. Alterations in lipid metabolism and consequently lipid composition have important therapeutic implications, as they affect the survival, membrane dynamics, and therapeutic responses of cancer cells^25–27^. Although the regulation of lipid metabolism in cancer remains unclear, a previous study described ACS activity in cancer cells. ACSs convert long-chain fatty acids to acyl-CoA. This reaction is a critical step in several lipid metabolic pathways, including phospholipid biosynthesis, lipid modification of cellular proteins, and β-oxidation^23,28^. ACSs are overexpressed in a variety of cancers^29–36^. Mashima et al. identified an ACS inhibitor as a tumor-selective inducer of apoptosis^22,23^. In this study, triacsin C did not induce cytotoxicity in PC-3 cells, but it remarkably affected porphyrin metabolism.

Microarray analysis revealed the metabolic changes in dormant cancer cells. The cell cycle is strongly repressed in dormant cancer cells, leading to resistance to drugs that target rapid cell proliferation. Contrarily, heme-related pathways and protein synthesis-related pathways are upregulated. We previously reported that dormant cancer cells accumulate heme at higher levels than non-dormant cancer cells in the absence of ALA exposure^16^. In addition, dormant cancer cells exhibit lower glucose uptake than non-dormant cancer cells^2^. Because the enzyme cytochrome c oxidase (complex IV), which is also known as a hemoprotein, plays a key role at the end of the electron transport chain^37,38^, we speculated that dormant cancer cells switch their metabolic pathway from highly glycolytic ATP synthesis (i.e., Warburg effect) to oxidative phosphorylation-related ATP synthesis by mitochondria. Many anticancer drugs target Warburg effect-related pathways, which might be one reason for tumor recurrence. Triacsin C represses the TCA cycle by inhibiting ACSs and mitochondrial activity including oxidative phosphorylation-related ATP synthesis^26^; therefore, PpIX accumulation after ALA treatment may be reduced.

ALA-PDT is one of the most promising forms of photodynamic therapy for clinical cancer treatment. ALA-PDT is well suited to the treatment of early-stage malignancies, as it produces a superficial effect that preserves the structure and function of underlying and adjacent tissues. The limitations of its use include its restriction to superficial cancers, a lack of efficacy in hypoxic regions^39^, and the need for interstitial optic fibers for nodular lesions^40^. Although clinical studies of dermatological, urological, gastroenterological, and gynecological cancers have been widely performed in several countries^40,41^, there are no established predictors of ALA-PDT efficacy. Our previous reports indicated that PEPT1 and ABCG2 are key transporters for intracellular PpIX accumulation^42^. Other reports identified ABCB1 as the key transporter that regulates the effects of PpIX^43^. In this study, we demonstrated that lipid metabolism including ACS expression is positively related to PpIX accumulation. These findings suggest that lipid metabolism in tumors may be predictive of ALA-PDT efficacy. Biopsy is a standard method for confirming cancer cells in tumors^44,45^. Immunostaining of ACSs or other lipid metabolism-related gene in biopsied specimens could have predictive utility. We plan to conduct further research to investigate this hypothesis.

The results of this study identified lipid metabolism upregulation as a key factor of the enhancement of PpIX accumulation in dormant cancer cells. Although lipid metabolism is considered a key pathway for cancer proliferation, this is the first report to demonstrate that lipid metabolism induces PpIX accumulation after ALA treatment. Moreover, we will continue to investigate other pathways involved in porphyrin metabolism using microarray analysis. Further study should reveal the mechanism and key regulators of PpIX accumulation in cancer cells.

## Supplementary Information

Supplementary Information 1.

Supplementary Information 2.

## Data Availability

The datasets generated during and/or analyzed during the current study are available from the corresponding author on reasonable request.
