# Supplementary material for: Enhanced lipid metabolism induces the sensitivity of dormant cancer cells to 5-aminolevulinic acid-based photodynamic therapy
Source: Sci Rep. 2021 Mar 31;11:7290. doi: 10.1038/s41598-021-86886-9 (PMC8012701; doi:10.1038/s41598-021-86886-9)
Supplement: Supplementary file 1 — Supplementary Information 1. [file 41598_2021_86886_MOESM1_ESM.docx]

# **Supplementary Information**

**Enhanced lipid metabolism induces the sensitivity of dormant cancer cells to 5-aminolevulinic acid-based photodynamic therapy**

**Taku Nakayama^a,b^*, Tomonori Sano^c^, Yoshiki Oshimo^c,d^, Chiaki Kawada^e^, Moe Kasai^b^, Shinkuro Yamamoto^a,e^, Hideo Fukuhara^a,e^, Keiji Inoue^a,e^, Shun-ichiro Ogura^a,b^**

^a^Center for Photodynamic Medicine, Kochi Medical School, Kohasu, Oko-cho, Nankoku-shi, Kochi, 783-8505, Japan

^b^School of Life Science and Technology, Tokyo Institute of Technology, 4259 Nagatsuta-cho, Midori-ku, Yokohama, Kanagawa, 226-8501, Japan

^c^Kochi Medical School, Kohasu, Oko-cho, Nankoku-shi, Kochi, 783-8505, Japan

^d^Kitano Hospital, Tazuke Kofukai Medical Research Institute

^e^Department of Urology, Kochi Medical School, Kohasu, Oko-cho, Nankoku-shi, Kochi, 783-8505, Japan

* Corresponding author

Taku Nakayama: [Taku.nakayama@kochi-u.ac.jp](mailto:Taku.nakayama@kochi-u.ac.jp), [taku.nakayama222@gmail.com](mailto:taku.nakayama222@gmail.com)

# **Supplementary Table S1**

All genes with changes in gene expression in 2D and 3D culture.

All analyses were conducted using Transcriptome Analysis Console ver. 4.0.1.36. Genes with changes in expression exceeding 1.5-fold and significant at p < 0.05 genes were extracted.
